# Bond Strength of Porcelain to Milled Sintered and Casting Base Metal Alloys

**DOI:** 10.30476/DENTJODS.2020.84347.1076

**Published:** 2021-03

**Authors:** Zahra Mohammadi, Meysam Mahabadi, Gholamreza Tabbakhian, Mahmud Talaakoob

**Affiliations:** 1 Postgraduate Student, Dept. of Prosthetic Dentistry, Faculty of Dentistry, Isfahan Islamic Azad University, Khorasgan Branch, Isfahan, Ira; 2 Dept. of Prosthetic Dentistry, Faculty of Dentistry, Isfahan Islamic Azad University, Khorasgan Branch, Isfahan, Ira; 3 Dental Technician, Master of Science, Faculty of Dentistry, Isfahan Islamic Azad University, Khorasgan Branch, Isfahan, Iran

**Keywords:** Bond strength, CAD-CAM, Casting, Metal-ceramic, Milled sintered alloy

## Abstract

**Statement of the Problem:**

The success of metal-ceramic restorations depends on the bond strength between porcelain and alloy. These restorations can be fabricated through different casting and computer-aided design/computer-aided manufacturing (CAD/CAM) techniques.

**Purpose:**

This study aimed to compare the bond strength of porcelain to milled sintered (Sintron) and casting (Co-Cr and Ni-Cr) base metal alloys.

**Materials and Method:**

In this *in vitro* experimental study, 63 rectangular bars (25×3×0.5 mm) were fabricated of three base metal alloys: casting Ni-Cr, casting Co-Cr, and milled sintered Co-Cr alloy. Feldspathic porcelain (3×8 mm) was applied at the center of each bar with 1.5 mm thickness. The specimens were thermally aged. Bond strength was evaluated through three-point flexural test. Failure mode was evaluated by optical and electron microscope. Data were analyzed with one-way ANOVA and Tukey's post hoc test (α=0.05).

**Results:**

The mean flexural bond strength of porcelain to milled sintered Co-Cr alloy (24.58±5.16 MPa) was significantly higher than that of casting Ni-Cr (21.13±6.34 MPa) (*p=* 0.03) and casting Co-Cr (20.98±4.84 MPa) alloys (*p=* 0.04). However, the two casting alloys were not significantly different in this regard (*p=* 0.93). The failure mode in all specimens was of cohesive type.

**Conclusion:**

Bond strength of CAD/CAM milled sintered Co-Cr alloy was better than that of the conventional casting alloys and could serve as a suitable alternative to those alloys.

## Introduction

Despite the technological advances of all-ceramic restorations, metal-ceramic restorations still play an important role in dentistry [ 1 ]. Strong bond of the metal-ceramic interface, which is the most susceptible area for cracking, is the major prerequisite for durability of metal-ceramic restorations [ 2 ]. Development of various computer-aided design/computer-aided manufacturing (CAD/CAM) systems has increased the quality of full-crown restorations, allowed fabrication of wax patterns by using castable materials, and eliminated numerous limitations of the conventional waxing technique [ 3 ].

Base metal alloys can be processed with CAD/CAM through two different approaches. These include additive method, which includes laser sintering, and subtractive method, in which materials of maximum strength are machine-milled. However, subtractive method is costly and only few CAD/CAM laboratory systems are able to process hard presintered cobalt-chromium (Co-Cr) blocks. The newly-developed soft non-presintered Co-Cr alloy (Sintron) can be processed in milling machines at reduced cost and time, and processing steps of quite comparable to non-presintered zirconia. 

Soft Co-Cr blank is processed in a material pre-state by dry milling. The material contains adhesive agents like organic binders and is milled in a green state. Then, to achieve full density, the milled structure is sintered at high temperature in an argon gas atmosphere, which slightly decreases the material volume [ 4 ]. The final density is affected by the sintering temperature and time [ 5 ]. Non-presintered alloys and their bond to porcelain have been limitedly investigated. Therefore, this study was designed to compare the bond strength of porcelain to non-presintered alloys (CAD/CAM milled sintered Co-Cr) and conventional casting alloys (casting nickel-chromium and Co-Cr). The study also aimed to evaluate the failure mode (within the porcelain or within the metal-porcelain interface), to help future studies address porcelain failure problems. The null hypothesis was that no difference exists between the non-presintered and casting alloys neither in their bond strength to ceramic nor in their failure pattern. 

## Materials and Method

In this experimental *in vitro* study, three groups of specimens (n=21 per group) were fabricated from non-presintered CAD/CAM milled Co-Cr (Sintron) (Ceramill Sintron, MS: solid state sintered, Amann Girrbach, Austria), casting Co-Cr (Wirobond sg, Bego Inc., USA), and casting Ni-Cr (Supremcast V; American Dent-All Inc., CA, USA). To fabricate Sintron specimens, 21 cubic specimens were designed and milled in green state to achieve the final dimensions of 25×3×0.5 mm, considering the post-sintering shrinkage. Having confirmed their identical size, the specimens were sintered under argon gas according to the manufacturers' instruction.

Casting Co-Cr specimens were designed with CAD/ CAM and milled out of polymethyl methacrylate (PMMA) blanks (PMMA Ceramill; Amann Girrbach AG, Austria) to reach the final dimensions of 25×3×0.5 mm. Then, they were placed in phosphate-bonded investment and heated according to the manufacturer's instructions. Having eliminated the acrylic residual, the molten alloy was cast into the investment via an induction casting machine. The specimens were bench cooled, then, sandblasted with 110-μm aluminum oxide particles and all sized to the desired dimensions by using a disk. Using the same method, casting Ni-Cr specimens were prepared and cast according to the manufacturer's instructions. 

**Figure 1:**
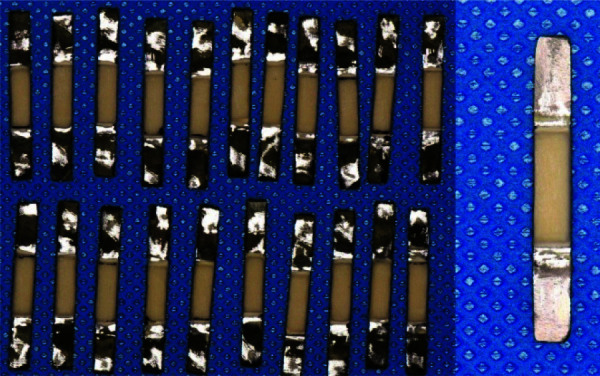
Veneered specimens

The middle one-third of non-presintered Co-Cr specimens was prepared according to the manufacturer's instructions through sandblasting with 110-µm aluminum oxide particles, cleansed with ultrasonic and steam cleaning. Then, bonding agent (Bredent GmbH & Co. KG, Germany) was applied, and veneered with porcelain (Creation CC, Creation Willi Geller International, Meiningen, Austria) (Figure 1). 

Casting Co-Cr specimens were treated on the middle one-third through sandblasting, ultrasonic and steam cleaning, respectively. Then, bonder was applied, followed by porcelain. Casting Ni-Cr specimens were sandblasted, ultrasonic and steam cleaned; and then, degassed. Porcelain veneering was done after applying the bonding agent according to the manufacturer's instruction. Silicone index was used to control the thickness and length of porcelain.

**Figure 2:**
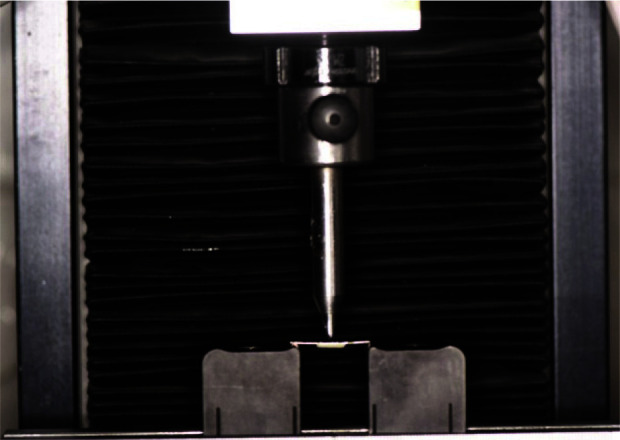
Universal testing machine

The specimens were all subjected to 5000 thermal cycles in water baths of 5-55 °C with a dwell time of 20 seconds per bath, a rest time of 5 seconds, and frequency of 1 cycle per minute. To assess the metal-porcelain bond strength with three-point flexural test as recommended by the ISO9693:1999(E) [ 6 ], the specimens were mounted on the universal testing machine with 20 mm distance between the leverages (Figure 2) with the veneer surface facing downward. 

**Figure 3:**
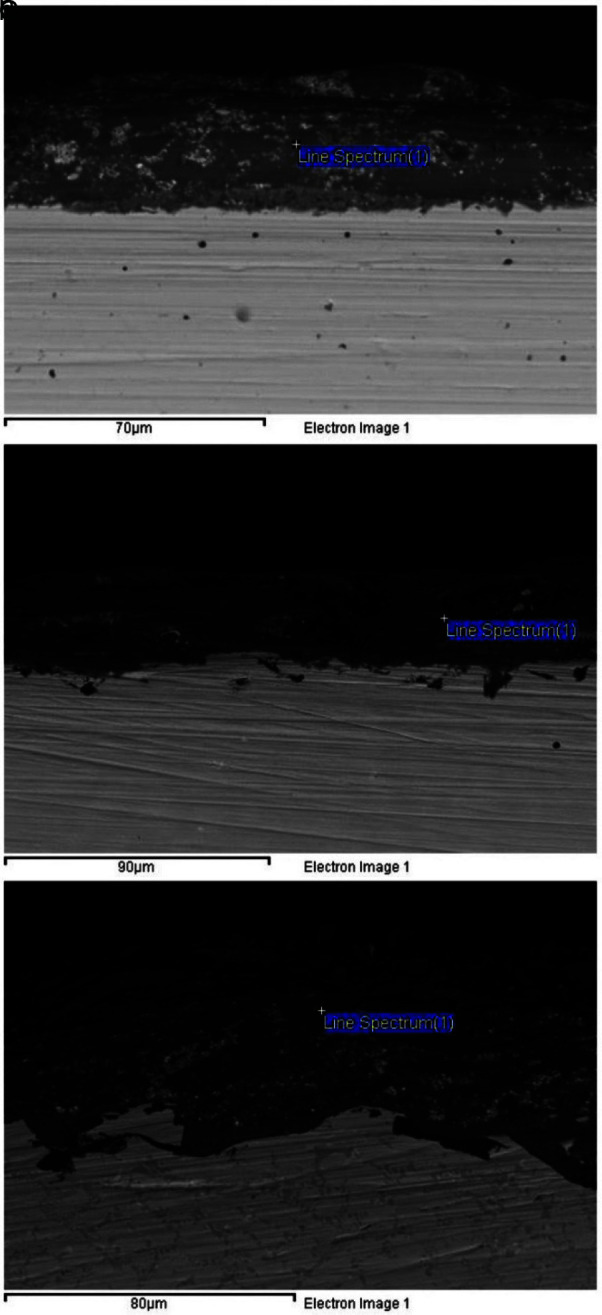
Debonded surface of specimen (×10.5): **a:** Sintron, **b:** casting Co-Cr, **c:** casting Ni-Cr

**Figure 4:**
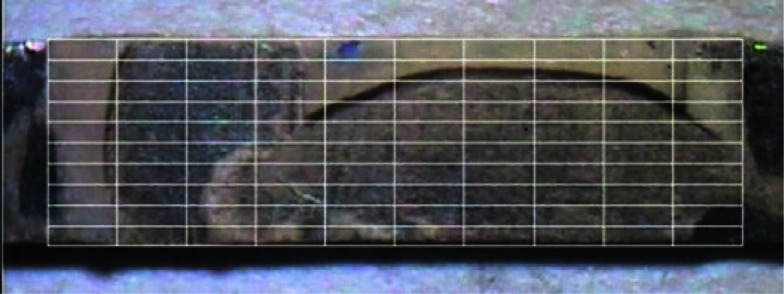
Determining the metal-porcelain failure mode

By using the designed pin, force was applied with a pyramidal tip and rectangular cross-section at a crosshead speed of 1 mm/min. The force leading to failure was recorded and the bond strength was measured in MPa by using $\frac{\mathrm{..3FL..}}{\mathrm{2bh 2}}$ formula. The metal-porcelain failure was scanned with scanner and images were assessed with digitalized computer at ×10.5 magnification (Figure 3). To determine the failure mode, each image was divided into 100 equal sections (Figure 4). If more than 50% of the squares were covered with porcelain, failure mode was considered as cohesive type; and if porcelain covered less than 50% of the area, it was considered as adhesive failure.

Moreover, to determine the failure percent of opaque-body in cohesive failures, if the remaining porcelain was >0.1 mm thick, failure was considered to be in the body, and if the porcelain layer was <0.1 mm thick, failure was considered to have occurred in the opaque layer. 

**Figure 5:**
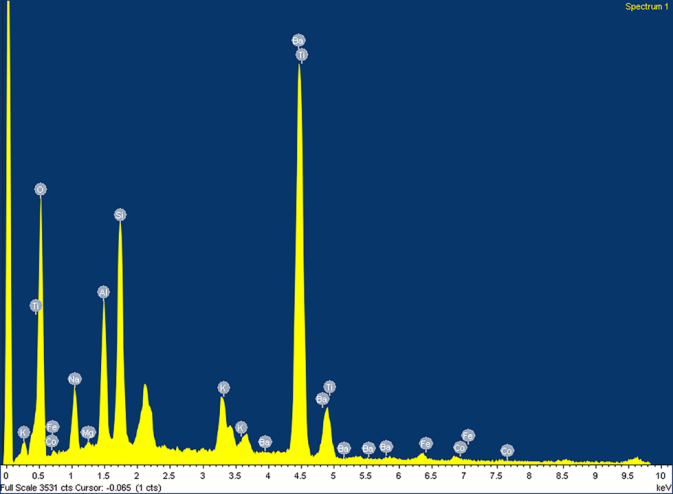
Energy dispersive x-ray spectroscopy of Sintron debonded surface

**Figure 6:**
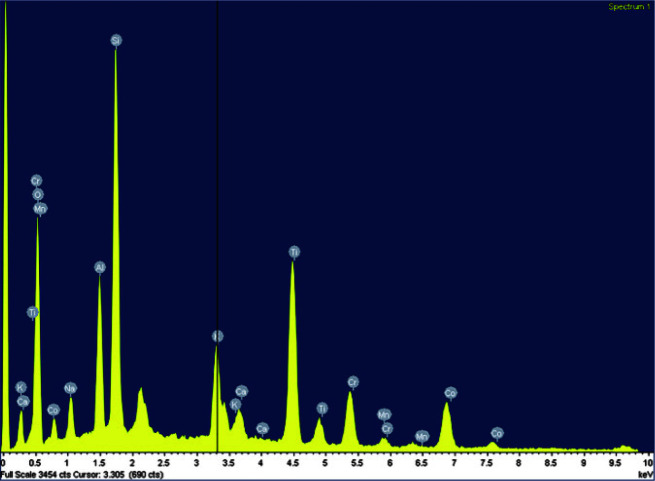
Energy dispersive x-ray spectroscopy of casting Co-Cr debonded surface

**Figure 7:**
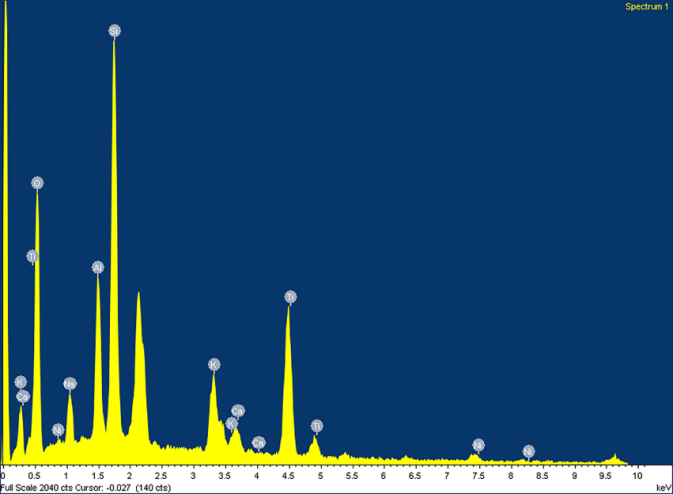
Energy dispersive x-ray spectroscopy of casting Ni-Cr debonded surface

After failure and debonding of porcelain, one specimen was selected from each group, gold-coated, and inspected with secondary electron and, back scattered detectors of electron microscope. For structural analysis, one specimen of each group was longitudinally cut to expose the cross-section and mounted in epoxy resin. The surface was covered with carbon sheet and examined through energy dispersive x-ray spectroscopy (EDX; Oxford Instrument, Oxfordshire, United Kingdom). Based on images, the surface properties of the specimens, metal-ceramic bond strength, and the composing elements were identified (Figures 5, 6 and 7). 

Data were analyzed by using SPSS software (version 24, SPSS Inc., IL, USA). Kolmogorov-Smirnov test was used to assess the normal distribution. One-way ANOVA was employed to compare the bond strength among the three groups. Tukey's post hoc test was used for pairwise comparison of the alloys in terms of bond strength. *p*< 0.05 was considered to be statistically significant.

## Results

The Kolmogorov-Smirnov test revealed that porcelain bond strength and the cohesive failure percentage in the body and opaque layers was normally distributed in all the three alloys. Therefore, one-way ANOVA was performed to compare these three variables among the three alloys. Only cohesive failure mode was seen in all the three alloys. Based on the results of one-way ANOVA, the mean bond strength of porcelain was significantly different among the three alloys (*p*= 0.03). Tuke-y's post hoc test revealed that the mean bond strength of porcelain to Sintron alloy was significantly higher than that to Co-Cr (*p*= 0.03) and Ni-Cr alloy (*p*= 0.04). But, no significant difference existed between Co-Cr and Ni-Cr base metal alloys in this regard (*p*= 0.93) (Table 1). One-way ANOVA also showed the three alloys to be significantly different in terms of the mean percentage of cohesive failure in the opaque layer (*p*= 0.03). According to Tukey's post hoc test, the mean percentage of cohesive failure in the opaque layer in Ni-Cr group was significantly higher than that in Co-Cr (*p*= 0.003) and Sintron alloy (*p*= 0.04). However, Co-Cr and Sintron base metal alloys were not significantly different in this regard (*p*= 0.32). The mean percentage of cohesive failure in the body layer was significantly different among the three alloys (*p*= 0.009); being significantly lower in Ni-Cr alloy than in Co-Cr (*p*= 0.002) and Sintron (*p*= 0.03). Yet, Co-Cr and Sintron alloys were not significantly different in this regard (*p*= 0.22) (Table 2). 

**Table 1:** Mean flexural bond strength of porcelain to the three alloys

Alloy	Mean	Standard deviation	*p* Value
Co-Cr	20.98	4.84	0.03
Ni-Cr	21.13	6.34	
Sintron	24.58	5.16	

**Table 2:** Mean percentage of cohesive failure in the body and opaque layers of the three alloys

Layer	Alloy	Mean	Standard deviation	*p* Value
Opaque layer	Co-Cr	75.24	8.04	0.01
	Ni-Cr	82	7.02	
	Sintron	77.43	6.03	
Body layer	Co-Cr	24.76	8.04	0.009
	Ni-Cr	18	7.02	
	Sintron	22.09	5.45	

As presented in Figures 5-7, energy dispersive x-ray spectroscopy analysis of the debonded surface of specimens revealed that Silicon (Si) was the most frequently detected element on the debonded surface.

## Discussion

The null hypothesis was partially rejected since metal-ceramic bond strength in non-presintered Co-Cr alloy was significantly higher than that in Co-Cr and Ni-Cr. However, all failures had cohesive pattern within the porcelain, indicating proper metal-ceramic bond strength. In line with the present study, Juntavee and Oeng's [ 7 ] detected similar findings regarding metal-ceramic bond strength. However, they reported all fractures to be of adhesive type in visual examination via microscope; whereas, in the present study, all fractures were cohesive as approved by the optical and electron microscopic images and energy dispersive x-ray spectroscopy of the debonding surface and the fractures cross-section.

The scanning electron microscopy images in Juntavee's [ 7 ] study showed micro-gaps in the metal-ceramic interface in the casting group; while no porosity was seen on the cross-section of specimens of the present study. Given the use of bonding in the present study, differences in the failure mode and fractured area can be attributed to the preparation method before applying the porcelain and the type of performed test.

Stawarczyk *et al*. [ 8 ] evaluated the bond strength of Ceramill Sintron ceramics with casting Co-Cr (Girobond NB) and laser-sintered Co-Cr alloy (Ceramill NP L) with different ceramics. With Creation ceramics, the bond strength of Sintron was higher than that of the cast alloy; which is consistent with the present findings. In the present study, the three tested alloys had similar elasticity coefficient; therefore, the formula ..3FL..2bh 2 was used to express the bond strength in megapascal. However, based on ISO 9693:2012, there is a special algorithm for alloys with different elasticity coefficient, which converts the failure force in three-point flexural test to megapascal with respect to the width and constant coefficients based on the elasticity coefficient [ 9 ].

Lee *et al*. [ 10 ] investigated the metal-porcelain shear bond strength, and found the shear bond strength of Sintron to be comparable or greater than the casting alloy (4 all). They reported mixed failure mode, which was not analyzed for surface elements based on surface images. In the present study, besides the interpretation of surface images, the cross-sectional image and the debonded surface characteristics were also analyzed, which revealed Silicon (Si) as the most frequently present element on the surface, confirmed the cohesive failure in all three groups. Silicon is the chief element in dental porcelain, which is not generally found in dental alloys, unless contaminated with investments or polishing abrasives used for surface preparation [ 11 ].

Marques de Melo *et al*. [ 12 ] investigated the bond strength of four base metal alloys (two Ni-Cr alloys and two Co-Cr alloys) to ceramics, and found no significant difference among them. Similarly, Ahmadzadeh and Ghanavati [ 13 ] reported that although Sintron bond strength was lower than Wirobond alloy, it was within the clinically acceptable range. Such a minor difference with the present study might be due to the different design of tests and specimens of the two studies. 

In De Melo's study [ 12 ], all the four groups (Wiron-99, 4all, Argeloy NP, and IPs d) had cohesive fracture within the porcelain, which is the most favorable type of failure according to Obrien's study [ 14 ]. In the current study, all the three alloys similarly had cohesive failures, only the fracture layer was different (opaque or body); cohesive fractures in the opaque layer of Ni-Cr base metal were significantly more than those in casting Co-Cr and Sintron alloys. According to the images of the specimens' cross-section, the border of the oxide and porcelain regions in the nickel-chromium group was much clearer, indicating that the failure occurred in this region. The mean percentage of failures in the opaque layer of cobalt-chromium and Sintron was not significantly different, despite their partly similar element composition (only different preparation techniques). 

As the well-known base metal alloys, Co-Cr and Ni-Cr have distinct reactions with ceramics [ 8 ]. The elements displaced at the metal-ceramic interface accounts for the chemical bond. In the present study, Supremcast V was used as the Ni-Cr alloy, whose bond to porcelain was insignificantly stronger than that of the Wirobond Co-Cr alloy. The presence of nickel, chromium, molybdenum, beryllium and aluminum helps creating a strong chemical bond between this alloy and the ceramic. Nonetheless, due to biocompatibility and allergy issues of beryllium and nickel, this alloy is better to be cautiously used in high-risk cases. 

Fabrication of restorations with sinterable alloy and CAD/CAM includes fewer stages compared with casting alloy restorations, which reduces the probability of error. These restorations are also superior due to the uniformity of their composition at all stages, thanks to the different manufacturing method. In casting, the elements of molten alloy may not be uniformly cooled and change the physical properties of alloy. Furthermore, reactions are likely to occur between the molten metal and the investment material in the casting method. Contamination with wax or resin residues is also possible in the casting method, which affect the mechanical and biological properties of the alloy. All the above-mentioned problems can be avoided by using a non-presintered alloy. 

Accordingly, it can be concluded that the sintering process along with CAD/CAM milling used for Sintron has many advantages over the conventional casting method. Sintron is also superior to casting Co-Cr because of its less hardness, which facilitates polishing. Generally, mechanical properties of Sintron are comparable, and in some cases, superior to casting alloys [ 15 ]. 

Among the limitations of the present study was the size of specimens for design and milling, which should be designed by specific software. Further studies are suggested with different alloy and porcelain brands and assessing the failure type in a larger sample size by using energy dispersive x-ray analysis.

## Conclusion

With respect to the results of the present study, it can be concluded that besides its superiority in restoration fabrication stages, Sintron bond strength to porcelain is not only comparable to that of casting method, but also higher *in vitro*. Undoubtedly, dentistry is advancing towards digital approaches so as to minimize the manual workflows both in the clinic and laboratory. Sintron has facilitated the digital fabrication of metal-ceramic restorations. Given the acceptable strength of this alloy to porcelain and the favorable cohesive fracture type in all specimens, Sintron can be a good substitute for casting alloys if other properties of this alloy are approved.
